# Implementing High-Intensity Gait Training in Stroke Rehabilitation: A Real-World Pragmatic Approach

**DOI:** 10.3390/jcm14155409

**Published:** 2025-07-31

**Authors:** Jennifer L. Moore, Pia Krøll, Håvard Hansen Berg, Merethe B. Sinnes, Roger Arntsen, Chris E. Henderson, T. George Hornby, Stein Arne Rimehaug, Ingvild Lilleheie, Anders Orpana

**Affiliations:** 1 Regional Rehabilitation Knowledge Center, Sunnaas Rehabilitation Hospital, 1453 Nesodden, Norway; stein.arne.rimehaug@sunnaas.no (S.A.R.); ingvild.lilleheie@sunnaas.no (I.L.); 2 Institute for Knowledge Translation, Carmel, IN 46033, USA; chende@knowledgetranslation.org; 3 Skogli Health and Rehabilitation Center, 2614 Lillehammer, Norway; pia.kroll@skogli.no (P.K.); havardhansen.berg@skogli.no (H.H.B.); meretheb.sinnes@skogli.no (M.B.S.); roger.arntsen@skogli.no (R.A.); anders.orpana@skogli.no (A.O.); 4 School of Medicine, Indiana University, Indianapolis, IN 46254, USA; tghornby@iu.edu; 5 Department of Nursing and Health Sciences, University of South-Eastern Norway, 3045 Drammen, Norway

**Keywords:** stroke rehabilitation, implementation science, knowledge translation, translation science, biomedical, gait disorders, neurologic, physical therapy specialty

## Abstract

**Background**: High-intensity gait training (HIT) is an evidence-based intervention recommended for stroke rehabilitation; however, its implementation in routine practice is inconsistent. This study examined the real-world implementation of HIT in an inpatient rehabilitation setting in Norway, focusing on fidelity, barriers, and knowledge translation (KT) strategies. **Methods**: Using the Knowledge-to-Action (KTA) framework, HIT was implemented in three phases: pre-implementation, implementation, and competency. Fidelity metrics and coverage were assessed in 99 participants post-stroke. Barriers and facilitators were documented and categorized using the Consolidated Framework for Implementation Research. **Results**: HIT was delivered with improved fidelity during the implementation and competency phases, reflected by increased stepping and heart rate metrics. A coverage rate of 52% was achieved. Barriers evolved over time, beginning with logistical and knowledge challenges and shifting toward decision-making complexity. The KT interventions, developed collaboratively by clinicians and external facilitators, supported implementation. **Conclusions**: Structured pre-implementation planning, clinician engagement, and external facilitation enabled high-fidelity HIT implementation in a real-world setting. Pragmatic, context-sensitive strategies were critical to overcoming evolving barriers. Future research should examine scalable, adaptive KT strategies that balance theoretical guidance with clinical feasibility to sustain evidence-based practice in rehabilitation.

## 1. Introduction

The field of knowledge translation (KT) bridges the gap between research and clinical practice by providing methods for the systematic implementation of evidence-based practices into patient care [1]. When planning a KT project, it is critical to target a practice with robust evidence supporting its effectiveness and safety [1]. Such practices must be backed by a comprehensive body of high-quality research, typically including well-conducted randomized controlled trials and systematic reviews, which demonstrate consistent positive outcomes across various settings and populations.

Stroke rehabilitation has an extensive amount of literature, including national and international clinical practice guidelines, designed to standardize care and improve patient outcomes [2,3,4,5,6]. These guidelines recommend interventions that are supported by strong evidence, such as high-intensity gait training (HIT), which has demonstrated efficacy in improving gait outcomes for individuals undergoing stroke rehabilitation [3]. HIT involves task-specific walking while targeting high heart rates (≥70% of predicted maximum heart rate) and is delivered on the treadmill, overground, and on stairs [7]. While studies demonstrate that HIT is effective in improving gait and balance outcomes when compared to usual care in controlled laboratory settings [8], successful implementation in real-world rehabilitation settings depends on fidelity, or applying the intervention as designed or intended. The fidelity of HIT is commonly measured using metrics such as stepping activity, heart rate targets, and frequency. Inpatient rehabilitation implementation studies have also demonstrated improvements in HIT result in substantial improvements in patient outcomes, although these studies did not achieve the levels of fidelity demonstrated in the research [9,10,11]. Achieving fidelity in routine clinical practice remains a challenge. More research is needed to examine implementation strategies, barriers, and facilitators to ensure that HIT is delivered with high fidelity when implemented into stroke rehabilitation practice.

This study observed HIT implementation in an inpatient rehabilitation facility that provided care to individuals in the subacute and chronic phases post-stroke. The specific aims of this study were to (1) describe barriers encountered during the implementation of HIT; (2) identify strategies to implement HIT with fidelity; and (3) monitor fidelity metrics to identify changes in clinical practice.

## 2. Methods

### 2.1. Study Design and Setting

This observational study was conducted at Skogli Helse- og Rehabiliteringssenter AS (Skogli, Lillehammer, Norway), a private interdisciplinary rehabilitation center located in Lillehammer, Norway, in collaboration with the Regional Rehabilitation Knowledge Center (RKR). RKR is an integral part of the health system in Norway, focusing on promoting best practices in rehabilitation across the region. This center collaborates with rehabilitation centers to enhance the quality of care using evidence-based implementation strategies. Skogli participated as a site in a larger Norwegian multi-site implementation project, Focused Intensive Repetitive Step Training (FIRST), that aimed to implement HIT in nine facilities. While HIT was implemented at these facilities, RKR guided the implementation and ensured that the interdisciplinary teams had the necessary skills and knowledge to implement HIT effectively. In addition, RKR provided education and training for the clinicians on standardized assessments and HIT. While each site conducted their projects separately, the teams at each facility came together for in-person and online training, mentoring, and project meetings. They also shared experiences related to strategies to overcome local barriers.

Skogli maintains an average occupancy of 82 patients per day under a contract with the Helse Sør-Øst RHF (i.e., government funding). The Skogli interdisciplinary care team consists of physiotherapists (PTs), occupational therapists, nurses, medical doctors, social workers, psychologists, a vision specialist, and a speech therapistwho work collaboratively to provide comprehensive rehabilitation. The facility employs ~12 full-time PT positions that are shared between 13 clinicians. Rehabilitation services are provided to individuals who have experienced a stroke, with approximately 120 patients with stroke treated per year. Historically, the typical length of stay at the facility is 21 days for patients with chronic stroke and 28 days for patients with subacute stroke. The stroke team includes three physical therapists, equivalent to 1.7 full-time employees, dedicated to providing stroke rehabilitation.

Traditional stroke rehabilitation at Skogli, prior to the implementation of HIT, included functional tests that were conducted at admission and discharge, with clinicians individually selecting tests based on each patient’s functional level and clinician preferences. Standard testing protocols were not used and varied among the clinicians. Treatment interventions focused on a variety of therapeutic techniques tailored to individual patient needs. Key components included functional training and mobility techniques, strength training, range of motion exercises, and gait and balance training. For patients with motor deficits in the upper extremities, task-specific training was provided in collaboration with occupational therapists. The treatment interventions were selected by the treating therapist and varied among clinicians.

### 2.2. Participating Clinicians and Patients

Both clinicians treating patients with stroke and individuals undergoing stroke rehabilitation were included in the study. Full or part-time clinicians who treated patients receiving care for stroke rehabilitation were invited to participate. The participants undergoing post-stroke recovery were over 18 years of age and received inpatient rehabilitation for post-stroke functional deficits at the participating facility. They also had a physiotherapy goal of improving walking function. If patients had previously received HIT, either at Skogli or another FIRST project site, they were also excluded from data collection. Additionally, all participants were required to have the ability to provide informed consent, with the option for designated caregivers to provide consent on their behalf if necessary. Participants were excluded if they used instrumentation, such as a ventilator, that restricted walking activities. Patients with significant co-morbidities, including uncontrolled cardiovascular, metabolic, respiratory, infectious, or psychiatric disorders, or malignancies, were also excluded. Furthermore, individuals with a previous history of orthopedic or neurologic disorders that prevented them from walking more than 45 m before the stroke, such as an amputation or lower extremity fracture, were not eligible for the study.

Coverage, an important fidelity metric that assesses whether all patients who might benefit from HIT were offered the intervention, was monitored and documented by the leader of the stroke unit. While the FIRST project inclusion and exclusion criteria are listed above, some patients were not offered HIT for reasons such as prioritizing other rehabilitation goals (e.g., vision training, speech, fatigue, and pain management), referral to Skogli specifically for the constraint-induced movement therapy program, or medical instability as determined by the patient’s physician. If a patient had a short length of stay (typically <3 days) due to re-hospitalization, new-onset COVID diagnosis, or violating local rules, they were not offered HIT. Similarly, some patients who did not meet the FIRST project data collection criteria were offered HIT because they could benefit from the intervention.

### 2.3. Implementation Framework and Phases

**Figure 1:**
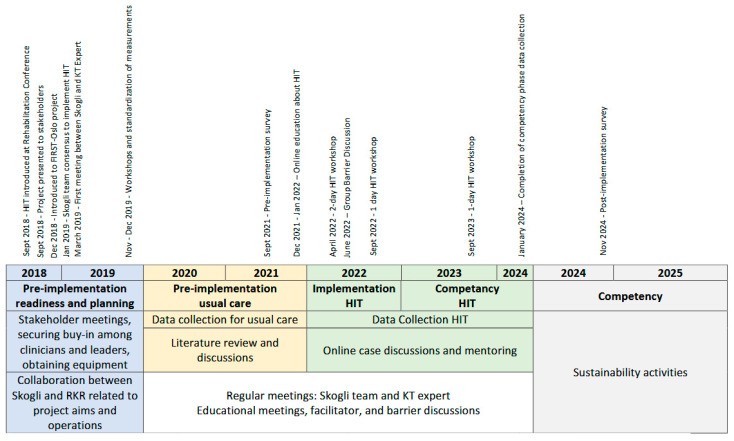
Timeline of pre-implementation, implementation, and competency activities.

The implementation plan was developed collaboratively by a KT expert from RKR and the interdisciplinary team at Skogli. The planning was guided by the Knowledge-to-Action (KTA) framework, which facilitated a systematic process for translating knowledge into practice [1]. The KTA includes two components, visually represented as the knowledge creation funnel (the generation of evidence and tailoring this evidence for implementation), and the action cycle, which includes seven phases to guide implementation processes. The seven phases are as follows: the selection of the practice to be implemented and assessing the know–do gap; adapting knowledge to the local context; assessing barriers and facilitators to knowledge use; selecting and using KT interventions (i.e., implementation strategies); monitoring knowledge use (i.e., fidelity); evaluating outcomes; and sustaining the implemented practice [1]. The project efforts were categorized into three phases: pre-implementation, implementation, and competency [12]. While a general timeline guided these activities, this was adjusted as needed to address the evolving demands of the project. The timeline and KTA plan are shown in Figure 1 and Table 1.

The pre-implementation phase followed the first four phases of the Knowledge-to-Action (KTA) framework, encompassing the identification of the know–do gap, the adaptation of evidence-based knowledge to the local context, the assessment of barriers and facilitators, and selection of targeted knowledge translation strategies. Discussions about implementing HIT were initiated by the leader of the stroke team, who was a PT who learned about HIT at a rehabilitation conference. Recognizing the need for a structured and evidence-based approach to gait training, the stroke team leader engaged the leader of the PT department, emphasizing HIT’s clinical benefits, alignment with evidence-based practice guidelines, and potential to enhance patient care and facility reputation. Together, they introduced the concept to the broader clinical team, facilitating discussions on feasibility, implementation challenges, and the potential impact on patient outcomes. These discussions highlighted HIT’s advantages, including standardized training parameters, greater consistency in care delivery, and opportunities for improved referrals and contractual relationships.

Following a consensus among clinicians and leadership, the leaders approached the Chief Executive Officer and positioned HIT as a strategic investment to modernize rehabilitation services. With leadership approval, Skogli joined the RKR-led FIRST project, gaining access to external facilitation, structured implementation support, clinician training, mentoring, and an implementation toolkit.

### 2.4. Pre-Implementation Phase: Monitoring Usual Care, Implementation of Standardized Assessments, and Fidelity Metrics

During the pre-implementation phase, we assessed the characteristics of usual care and implemented assessments to measure the patients’ functional level and rehabilitation outcomes. Heart rate was monitored using the OH1 (Polar Electro, Kempele, Finland) and a smartphone for recording. Fidelity metrics were gathered, including peak heart rate during PT sessions, not including sessions with >50% of the time spent on outcome measures, and the time patients spent at ≥70% of their maximum heart rate, calculated using Equation 211 − (0.64 × age) [13]. Borg’s Rating of Perceived Exertion (RPE) was also collected throughout PT sessions [14,15]. Stepping metrics were monitored, including steps per day, steps per PT session, stepping rate, and the number of minutes spent stepping using the StepWatch (Modus, Inc., Washington, DC, USA) [15]. These data were described as both absolute and relative values, where the absolute values described the number of minutes and the relative values referred to the percentage of the session. The fidelity metrics are defined in Table 2.

Measures implemented included gait speed, which was assessed using self-selected velocity (SSV) and fast velocity (FV) [2]. Walking distance was measured using the 6-Minute Walk Test (6MWT) using the instructions to “cover as much ground as possible over 6 min” [2]. Balance was assessed using the Berg Balance Scale (BBS), and for patients scoring above 50 on the admission BBS, the Mini-Balance Evaluation Systems Test (Mini-BESTest) was used [2]. These measures have excellent psychometric properties and are strongly recommended to be used in patients with stroke [2]. Clinicians were trained on standardized assessment protocols to ensure consistent data collection methods.

### 2.5. Implementation of HIT

After observing usual care practices to understand baseline rehabilitation characteristics and outcomes, the focus shifted to implementing HIT. The process emphasized selecting and applying tailored KT interventions to overcome barriers. A primary goal of the implementation plan was to deliver HIT with fidelity, meaning that it was delivered similarly to how it was studied [16]. The KT plan followed an iterative cycle of assessing barriers, selecting and using KT interventions, and monitoring knowledge use. If fidelity goals were not met, the cycle was repeated, allowing for continuous adjustments to optimize the integration of HIT into clinical practice.

HIT was recommended to be used in practice at a frequency of four times per week for one-hour sessions [8,15]. Recommendations also included delivering HIT at an intensity of 70% to 85% of maximum HR or 14–18 on the RPE scale (hard or very hard), with approximately 40% of each one-hour session spent in the target heart rate zone (70–85% of maximum heart rate). Fidelity metrics were monitored throughout the implementation phase to understand adherence to HIT recommendations. These are outlined above (see the pre-implementation phase). Annual feedback sessions provided clinicians with synthesized fidelity metrics, which facilitated ongoing efforts to improve fidelity. In addition, coverage was monitored to determine if all patients who may benefit from the intervention were offered it [16]. Each patient was screened for the potential to benefit from HIT, and information about why HIT was not offered was documented.

### 2.6. Barrier and Facilitator Identification

To understand barriers and facilitators, clinicians participated in informal discussions about barriers and facilitators to implementing standardized assessments and HIT in clinical practice. In addition, a designated PT systematically documented barriers encountered during the implementation phase, recording detailed information on the timing and nature of the barrier and strategies employed to overcome it. After the completion of the project, the team also reflected on and documented the facilitators of the implementation of HIT.

The barriers and facilitators identified during the project were systematically identified and categorized using the Consolidated Framework for Implementation Research (CFIR) [17]. Using its updated version, CFIR organizes these factors into five domains that describe the innovation (i.e., practice being implemented), outer setting (e.g., setting in which the rehabilitation center exists, such as the health system), inner setting (i.e., setting in which the practice is being implemented), individuals (i.e., roles and characteristics of individuals), and implementation process (activities and strategies used to implement the intervention). In addition, there are several constructs that are associated with each domain. Operational definitions of the domains and constructs are located on the CFIR website [18]. While the CFIR did not guide the selection of KT interventions, it is presented here as a method to describe the barriers and facilitators.

### 2.7. Selection of KT Interventions

Barriers encountered during the project were systematically identified and addressed through an iterative and collaborative process of developing KT interventions. Practical barriers, such as logistical challenges, were often resolved directly by clinicians, leveraging their detailed understanding of culture, daily operations, and team dynamics. However, more complex barriers required guidance from RKR’s KT expert and HIT content experts. The clinical team met routinely to discuss these challenges, allowing clinicians to share insights and propose potential solutions. This ongoing, adaptive process ensured that the strategies developed were practical and effective, enabling the team to respond dynamically to evolving needs. The KT interventions developed during the project are detailed in Table 3.

In addition to these tailored KT interventions, the team was provided with an implementation toolkit from RKR. This toolkit included resources such as online courses that teach clinicians and students about the practices, evidence-based protocols for measurement administration and HIT delivery, evidence-based resources that summarize measurement and HIT information, target heart rate calculators, clinical prediction rule calculators, gym and lanyard signs (e.g., rating of perceived exertion, heart rate zones, decision-support tools), and short videos on strategies to overcome barriers (e.g., ankle taping). In addition, the toolkit included implementation resources such as standardization instructions, data collection sheets, and screening forms. The team adapted these resources to meet their local needs.

### 2.8. Ethics Approval

The project was approved by the Southeastern Norway Ethics Committee (approval 2016/873), and all participants provided written informed consent.

### 2.9. Data Collection and Statistical Analysis

Data were grouped and analyzed by implementation phase. Normality was assessed using Shapiro–Wilk tests, and homogeneity of variances was evaluated with Levene’s test. Based on these assessments, appropriate parametric or non-parametric tests were applied. An independent samples Kruskal–Wallis test and the Kruskal–Wallis H statistic were used to compare the demographics and baseline function of patient groups. For stepping metrics, an Analysis of Covariance (ANCOVA) or Quade’s Non-parametric ANCOVA was used, adjusting for 6MWT distance as a covariate. Session times, number of sessions, HR metrics, and RPEs were analyzed without a covariate using one-way ANOVA or Kruskal–Wallis tests, depending on normality and variance assumptions. When significant group differences were identified, post hoc comparisons were conducted using Tukey’s HSD for parametric tests and Bonferroni-adjusted pairwise comparisons for non-parametric tests. Statistical analyses were conducted using IBM SPSS Statistics (Version 28, IBM Corp., Armonk, NY, USA) with significance set at α = 0.05.

To mitigate potential bias and ensure objectivity, data processing and analysis were conducted collaboratively by the lead author, a researcher is employed at Skogli, and the broader project team. Team members had access to the data and contributed to the interpretation of results through joint discussions. This collaborative approach ensured transparency, challenged assumptions, and reduced the risk of confirmation bias related to the lead author’s dual role as project facilitator and researcher.

## 3. Results

The project was divided into distinct phases over a seven-year period, where the timeline was adjusted to respond to the needs of the project and to facilitate successful implementation. The timeline for the project is shown in Figure 1.

Three clinicians contributed to the collection of data on 99 patients (n = 38 pre-implementation; n = 30 implementation; n = 31 competency). Participants with subacute (n = 40) and chronic stroke (n = 59) were recruited across the three phases. Table 4 provides an overview of clinician demographics, and Supplementary Material Table S1 describes patient demographics and baseline function. Participants with subacute stroke were similar across phases in terms of age, the time since stroke, and length of stay. The median age ranged from 71 to 73 years, and time since stroke at admission was consistent, with medians of approximately 40–56 days. Average gait speeds (SSV and FV) were not significantly different across phases. Among individuals with chronic stroke, baseline characteristics were also generally comparable across phases. The median age ranged from 65.5 to 71 years, and time since stroke varied more widely from ~450 to >1000 days. There were no statistically significant differences in self-selected or fast gait speed or FAC scores across the phases. While the baseline 6MWT was not statistically different between groups, the differences in 6 MWTs were near the threshold of the minimally important clinical difference of 65–70 m [19] and 50 m [20] for subacute and chronic stroke groups, respectively. Therefore, we utilized the 6MWT as a covariate for the stepping fidelity metric analysis for both groups.

### 3.1. Fidelity Metrics

A total of 271 patients were screened at admission. Of these, 91 were not offered HIT due to referral for other goals, such as the constraint-induced movement therapy program, 7 were not offered HIT due to medical instability, and 2 declined treatment. Thus, 167 patients could have benefited from HIT, and 87 received the intervention, resulting in a 52% coverage rate. While 87 patients received the intervention, only 61 are reported here from the implementation and competency phases due to data collection exclusions (see participants section). Detailed data related to coverage are presented in Table 5.

Patients with subacute stroke completed 14 to 17 sessions, and individuals with chronic stroke completed 12 to 14 sessions as part of their plan of care. For both groups, there were no significant differences in the total number of sessions across phases. After adjusting for admission 6MWT, average daily stepping did not significantly differ across implementation phases. However, steps per session, stepping duration, and stepping rate were significantly higher in the implementation and competency phases compared to the pre-implementation phase. No significant differences were observed between the implementation and competency phases for these stepping metrics. Similarly, heart rate and RPE metrics increased from pre-implementation to implementation and remained elevated in the competency phase.

In the subacute stroke group, fidelity metrics improved substantially across phases. Steps per session increased from a mean of 748 (435–1044) in the pre-implementation phase to 1853 (774–2195) during the implementation and 2306 (1492–2608) in the competency phase (*p* < 0.001). Minutes stepping per session also increased from 19 (6) minutes to 31 (11) and 37 (8) minutes, respectively (*p* < 0.001), and the stepping rate increased from 35 (9) to 49 (17) and 54 (12) steps per minute (*p* < 0.001). Time spent in the target heart rate (HR) zone increased from a median of 1 min (1% of session) in the pre-implementation phase to 18 min (40%) during implementation phase, and 20 min (45%) in the competency phase (*p* < 0.01). Similarly, time spent in the RPE zone increased from 2 min (5%) to 22 min (48%) and 21 min (34%) (*p* < 0.001). These fidelity gains occurred without significant increases in the total session time or number of sessions, suggesting improved quality of HIT delivery over time. For full fidelity metrics, please refer to Supplementary Material Table S2a.

For individuals with chronic stroke, fidelity also improved significantly. Steps per session increased from 584 (280 to 809) in the pre-implementation phase to 1803 (1130–2155) in the implementation phase and 2001 (1467–2552) in the competency phase (*p* < 0.001). Stepping rate improved from 32 (11) to 48 (12) and 52 (15) steps per minute (*p* < 0.001), and the number of minutes stepping increased from 16 (12 to 19) to 33 (26–37) and 38 (29–43) minutes (*p* < 0.001). Time in the target HR zone increased from 2 min (5% of session) to 17 min (31%) and 13 min (21%) across phases (*p* < 0.001). The RPE zone time also increased significantly from 3 min (6%) to 19 min (37%) and 20 min (36%) (*p* < 0.001). These improvements occurred despite similar session lengths and highlight enhanced adherence to HIT intensity targets, reflecting successful implementation over time. For full fidelity metrics, please refer to Supplementary Material Table S2b.

### 3.2. Barriers and Facilitators

The barriers and facilitators are shown in Table 3. Barriers were identified across the phases of the project. Early-stage barriers occurred during the pre-implementation phase and were related to the implementation of assessments and planning for HIT implementation. These barriers were spread across several CFIR domains and included inner setting, innovation, and individual characteristics. The barriers were related to learning testing procedures and interpretation, project-related processes, and securing equipment and space. As the clinicians began to learn about how to use HIT in clinical practice, the barriers mainly focused on individual characteristics. Specifically, they focused on the knowledge and skills related to delivering HIT in clinical practice. During this phase, barriers evolved to include the domain of the inner setting as the team recognized different equipment and space needs. In addition, the need to further adapt HIT for their practice was identified, which was a strategy that targeted a barrier categorized as innovation adaptability. Later in the implementation phase of HIT, the barriers continued to evolve. While they mainly focused on individuals, the barriers were described as a need to refine various aspects of clinical decision-making related to HIT.

The facilitators were spread across the inner setting, individuals, outer setting, innovation, and implementation process domains. Many facilitators focused on the inner setting and described a learning-centered culture, the alignment of the mission and priorities, and the connections among the clinicians and leaders. The individual facilitators were described as clinicians’ motivation and experience, and supportive leaders. The implementation process facilitators were related to the team initiating the project and their collaboration during implementation. The outer setting facilitators included partnerships and connections with professional associations, RKR, and other participating sites, and the belief that providing high-quality care could result in increased patient referrals. Lastly, the packaging of the HIT implementation program facilitated the project.

## 4. Discussion

This study demonstrates the real-world feasibility of HIT implementation in inpatient stroke rehabilitation, highlighting fidelity improvements, barriers, facilitators, and implementation strategies. Notably, improvements in HIT fidelity metrics (i.e., stepping and heart rate metrics) were observed, indicating that the intervention was delivered with increased fidelity. While these metrics are not as high as observed in research laboratories, they are aligned with fidelity metrics achieved in other inpatient rehabilitation implementation studies of patients who had a lower functional level at admission [8,9,10]. Although a structured implementation plan facilitated implementation fidelity improvements between 2022 and 2023, achieving high coverage levels in clinical practice remains challenging. Future implementation efforts should explore strategies to enhance coverage while maintaining clinical feasibility, including workflow adaptations, clinician training on patient selection, and system-level support to optimize HIT integration into routine rehabilitation.

The overall coverage rate was 52%; thus, approximately half of the patients who may have benefited from HIT received the intervention. The lower coverage observed in 2024 (29%) reflects a brief data collection window, as the project concluded in January and only 17 patients were admitted during that month. This contrasts with the 125+ patients admitted during 2022 and 2023 and likely underrepresents typical coverage trends due to normal fluctuations among patient admission rates. While the overall coverage rate is higher than that of another published Norwegian HIT implementation study [9], it is much lower than the ~96% coverage rate observed in a US study [10]. We did not systematically collect the goals of patients not enrolled in this project; however, notes from patient screening forms indicated that many patients were treated for other rehabilitation goals, such as fatigue management, cognitive rehabilitation, upper extremity function, or vision training. In addition, many of these patients had relatively high baseline functions and reported a perception of no difficulty with walking on a subjective assessment. This suggests that clinicians may have prioritized other areas of need based on patient goals and functional status. Recognizing and tracking these factors in future projects could support the development of a targeted implementation plan to improve coverage if needed.

The evolving nature of barriers encountered during HIT implementation in inpatient rehabilitation is demonstrated in this study. Early challenges centered on logistical issues and shifted toward optimizing HIT delivery as implementation progressed. Later, barriers focused on refining treatment, decision-making, and sustaining fidelity. Previous studies have described many barriers that arise during HIT implementation, such as resource constraints, clinician knowledge, and fidelity challenges [11,21]. While previous research identified the presence of barriers, this study uniquely documents their evolution over time. By illustrating their progression, this study highlights the need for adaptive and dynamic implementation strategies. Recognizing how barriers change over time allows teams to apply targeted strategies at different stages. Further research should investigate this progression across different healthcare settings and determine effective and efficient implementation strategies.

The implementation science literature emphasizes the importance of the pre-implementation phase in establishing a strong foundation for implementation with fidelity [22]. The pre-implementation phase in this project required 14 months and was marked by activities such as securing support from the leadership team and clinicians, understanding the HIT-related barriers and facilitators, and implementation planning. In a comparable HIT implementation project, clinicians spent approximately two years completing this phase, focusing on building consensus for HIT implementation and facilitating readiness for change [21]. Both studies included time for these activities to occur, which may have impacted the success of the project. Studies have demonstrated that comprehensive planning and completion of activities during the pre-implementation phase significantly predict implementation with fidelity and sustainment [22,23]. Furthermore, studies indicate that incomplete pre-implementation activities can undermine the effectiveness of later implementation efforts, as even a well-executed implementation phase cannot compensate for early gaps [22]. While implementation science has recognized the importance of pre-implementation, there is limited research on how to optimize pre-implementation activities. Future research should focus on identifying the most effective strategies for engaging stakeholders, addressing context-specific barriers, and designing a structured pre-implementation plan.

This project demonstrates a pragmatic, real-world implementation of evidence-based practices, illustrating how a practical and context-sensitive approach to KT has the potential to address the inherent complexities of clinical practice. While we used the KTA framework to plan and guide the overall implementation process and the CFIR to categorize barriers and facilitators, we did not use a theory to guide implementation strategy selection [24]. Instead, we took a pragmatic approach, leveraging the team’s understanding of the local clinical context and barriers to iteratively select and refine KT interventions. This approach allowed for flexibility in addressing emerging challenges in real time and ensured that strategies were directly relevant to clinicians’ workflow, resource availability, and patient needs. Although the literature often recommends frameworks such as the Theoretical Domains Framework [25] or the CFIR [24] to guide the selection of KT interventions, studies suggest that these approaches can sometimes be complex and difficult for clinicians without formal KT training to apply in real-world settings [24,26]. Notably, Graham and colleagues emphasize that both theory-driven and common-sense approaches have value in implementation and should be studied, particularly when tailored to the needs and capacities of frontline clinicians [1]. A pragmatic approach, such as the one used in this study, might be more intuitive and feasible for clinicians who are embedded in the clinical environment but have limited experience with implementation science. However, a potential trade-off is the risk of missing theoretically informed strategies that could enhance implementation success, particularly when barriers are complex and require multi-level interventions. Future research should explore how pragmatic approaches can be balanced with theory-driven methods to optimize implementation planning while maintaining feasibility and adaptability in practice.

External facilitation and participation in the larger FIRST project may have contributed to the implementation of HIT by providing structured guidance, external expertise, resources, and a sense of collective learning [27]. The RKR team played a key role in facilitating the implementation process by offering targeted training, structured feedback, and ongoing mentorship, which may enhance clinician engagement and sustain motivation [27]. External facilitation is a well-established implementation strategy that helps bridge knowledge gaps, sustain motivation, and support problem-solving by integrating both internal and external expertise [28,29]. It is particularly effective in complex interventions by fostering organizational learning and adapting strategies to fit the local context [30]. Additionally, external facilitators may play an important role in guiding teams through the implementation process and helping them navigate barriers and reinforcing accountability [31]. Skogli’s involvement in the larger FIRST project also allowed clinicians to connect with peers from other facilities, exchange knowledge, and collaboratively address common barriers. Research suggests that being part of a larger initiative strengthens professional identity, encourages shared problem-solving, and enhances accountability, all of which may have contributed to implementation success [32,33]. Furthermore, the availability of a structured implementation toolkit that was available to FIRST project sites included online courses, decision-support tools, and evidence-based support for clinicians in overcoming barriers related to resources. The local adaptation of these resources may have improved clinician engagement and self-efficacy, aligning with findings that highlight the importance of packaged resources and structured training in enhancing adherence to implemented practices [33,34,35]. These combined factors likely reinforced the successful integration of HIT into clinical practice, demonstrating the value of external facilitation, structured resources, and engagement in a broader implementation effort. Future research should explore how these factors interact and influence implementation over time to better understand their role in implementing evidence-based practices with fidelity.

### Limitations

While this study provides valuable insights into the real-world implementation of HIT in inpatient stroke rehabilitation, several limitations should be acknowledged. First, this was a single-site study within a broader multi-site implementation effort, which may limit the generalizability of the findings to other settings with different organizational structures, healthcare policies, or patient populations. While the larger FIRST project provided opportunities for cross-site learning, this study focused on implementation at one site, making it difficult to determine whether similar implementation strategies would be effective in other rehabilitation facilities with different resources and clinical workflows. Second, the pragmatic approach to selecting KT interventions, while contextually relevant and feasible for frontline clinicians, may have omitted theoretically driven strategies that could have further optimized implementation success. The absence of a structured framework to systematically map barriers to specific KT interventions presents a potential limitation, as it may have resulted in missed opportunities to apply more targeted, evidence-based strategies. Third, fidelity metrics were calculated using the entire duration of each therapy session, rather than isolating only the time actively spent delivering HIT. While this reflects how sessions are structured and delivered in practice, it may underestimate the fidelity achieved during the actual HIT portions. Although sessions with more than 50% of time spent on outcome measures were excluded, the inclusion of sessions with some testing may have influenced fidelity metrics by reducing the total time available for HIT. It is likely that the total fidelity to HIT principles, if calculated solely based on the active HIT time, could be higher. However, we did not have valid or consistent methods to isolate HIT time alone. Patient characteristics may have also influenced their ability to engage in the intervention, which could impact fidelity metrics. Although inclusion criteria and baseline functional measures were consistent across groups, variability in time since stroke occurrence in the chronic stroke group may have influenced participation and fidelity. Additionally, we did not directly measure patient motivation or readiness, which may have contributed to differences in implementation fidelity across phases.

Another limitation is the lack of comprehensive data collection on the reasons HIT was not offered to eligible patients. While many of those who were not offered HIT were noted as pursuing alternative rehabilitation goals, which were often aligned with high baseline functional levels, this information was not consistently documented. Similarly, systemic constraints such as therapist availability or vacation schedules were not tracked, limiting our ability to assess their influence on HIT coverage. Future research should incorporate the structured documentation of these factors to better understand and address factors contributing to the underutilization of evidence-based interventions.

## 5. Conclusions

This study demonstrates the feasibility of implementing HIT in inpatient stroke rehabilitation, highlighting the importance of structured pre-implementation planning, clinician engagement, and external facilitation. These findings underscore the evolving nature of implementation barriers and the need for adaptive, pragmatic knowledge translation strategies to sustain fidelity in clinical practice. Research is needed to identify the most impactful implementation strategies for optimizing fidelity and to evaluate scalable, pragmatic approaches that balance theory-driven and clinician-led implementation methods across diverse rehabilitation settings.

## Figures and Tables

**Table 1:** Knowledge translation plan and results.

Pre-Implementation Phase Activities		
Activity	Description	Results
Education about HIT	The stroke team leader attended a regional rehabilitation conference and learned about HIT in an educational session	Team leaders were interested in learning more about HIT and possibly implementing HIT in practice
Engage leaders	Meetings between the stroke team leader, the PT department leader, and executives (CEO/CFO) about the benefits of HIT	Organizational leaders endorsed and supported the implementation of HIT
Engage clinical team	The stroke and PT leaders presented the potential HIT implementation project to the clinical team. Discussions included HIT feasibility, supporting evidence, and potential implications of using HIT.	The clinical team came to a consensus and agreed to implement HIT in stroke rehabilitation
KTA Phase during Pre-Implementation	Methods for Each Phase	Results
Phase 1: Identify Problem, Determine the Know–Do Gap, Identify, Review, and Select Knowledge	Informal discussions: Clinicians and managers reviewed evidence and selected a specific HIT protocol for implementation	Current practice is described as including several interventions to address gait-related impairments; Selected HIT protocol as described by Holleran et al., 2014 [7]
Phase 2: Adapt Knowledge to Local Context	Reviewed current evidence and dosages of HIT; Adaptation of the research protocol to fit into the local context	Local adaptations for HIT; Adjusted inclusion/exclusion criteria to include walking impairments and associated goals; Translated and adapted data collection forms
Phase 3: Assess Barriers and Facilitators for Knowledge Use	Documentation of observed barriers; Informal interviews with clinicians and managers; An iterative process of barrier and facilitator assessment, the implementation of KT intervention, and monitoring	Barriers included intervention adaptability and costs, available resources, compatibility, culture, individual stages of change, and knowledge and beliefs; Barriers evolved over time from many CFIR domains to focus on individuals
Phase 4a: Select and Tailor KT Interventions	KT interventions were designed to target barriers; Design of KT interventions co-developed by the clinician and research team	A multi-component KT intervention was designed that evolved over time to target the high-priority barriers
KTA Phase—Implementation	Methods for Each Phase	Results
Phase 4b: Implement KT Interventions	KT Interventions used to target identified barriers	An iterative approach to assessing barriers, using KT interventions, and monitoring the use of HIT
Phase 5: Monitor Knowledge Use	Collected stepping activity and assessed the amount of time spent in the HR/RPE zone; Informally reviewed treatments during case discussions	An iterative approach to assessing barriers, using KT interventions, and monitoring the use of HIT until stepping and HR monitoring indicate fidelity was achieved
KTA Phase—Competency	Methods for Each Phase	Results
Phase 6: Evaluate Outcomes	Provider level: Surveys and focus groups on clinician attitudes, perceptions, and perceived adherence to recommendations; Patient level: Comparison of results from the three phases	Data collection and analysis are currently underway and, therefore, not reported in this manuscript
Phase 7: Sustain Knowledge Use	Updated local PT guidelines incorporating HIT as a standard treatment for stroke; Continued regularly scheduled meetings for discussions on clinical reasoning in HIT; Continued collection and analysis of fidelity metrics and outcome measures	Data collection is currently underway and, therefore, not reported in this manuscript

**Table 2:** Definitions of terms used in fidelity calculations.

Term	Definition	Calculation
Coverage	The percentage of eligible patients who were offered high-intensity gait training (HIT).	(Number of patients offered HIT ÷ Number of eligible patients) × 100
Average daily stepping	Number of steps taken per weekday during the rehabilitation stay.	Total number of steps, counted only during the weekdays
Steps per session	Number of steps taken during each physiotherapy (PT) session.	Total steps taken during PT sessions
Min stepping per session	Number of minutes spent stepping during each session.	Total minutes with ≥10 steps/min during PT sessions
Average stepping rate	Number of steps per minute during stepping time in each session.	Mean stepping rate for minutes with at least 10 steps during PT sessions
Steps for 60 min sessions	Equivalent number of steps taken if the session was 60 min in duration.	Steps during PT session/session duration (min) × 60 (min)
Minutes stepping for 60 min sessions	Equivalent number of minutes stepping if the session was 60 min in duration.	Min stepping during PT session/session duration (min) × 60 (min)
Session duration	Total time scheduled for the physical therapy session.	Average or median of documented minutes the patient was scheduled for PT
HR max	Maximum heart rate achieved during the session.	Average or median of highest recorded HR during session
HR avg	Average heart rate recorded during the session.	Average HR over all PT sessions
Total time in HR zone	Total minutes spent at ≥70% HRmax	Average or median of the total minutes in target zone per session
% session in the target HR zone	Percentage of the therapy session spent at ≥70% HRmax	(Minutes in HR zone ÷ session duration) × 100%
RPE max	Highest perceived exertion rating reported during the session (scale 6–20).	Average or median of highest RPE score reported
Total time in RPE zone	Total minutes at RPE ≥ 14.	Total minutes RPE ≥ 14 per session
% session in the target RPE zone	Percentage of the scheduled therapy time spent at RPE ≥ 14.	(Minutes in RPE zone ÷ session duration) × 100%

**Table 3:** Barriers and facilitators observed throughout the project.

Pre-Implementation 2020–2021 Measuring Characteristics of Care and Outcomes and Preparation for Implementing HIT				
Month/Year	CFIR Domain	CFIR Construct	Barrier Description	Implementation Strategy
December 2019	Individual Characteristics	Capability	Variation in administration procedures for outcome measures across therapists	Conducted meetings with therapists to standardize test protocols. Created a testing station with standard equipment and scheduled bi-annual practical testing within the PT team. PTs completed a course on the use of measurement tools and outcomes in rehabilitation offered by RKR
January 2020	Inner Setting	Materials and Equipment	Necessary equipment for safe gait training	Acquired equipment, including a treadmill with harness system, a rail for harness-assisted overground walking and blood pressure (BP) and heart rate (HR) monitors for each PT
October 2021	Innovation	Innovation Design	Difficulty carrying essential items during treatments (e.g., HR monitors, BP cuffs, and documentation tools)	Introduced the use of fanny packs, enabling hands-free access to equipment and patients. Each PT was provided with personal BP and HR monitors
October 2021	Innovation	Innovation Complexity	StepWatch placed wrong or forgotten	Created a poster to illustrate instructions in patient rooms and nursing offices. Reminders were included in nurses’ daily checklist and the treatment protocol of PTs
November 2021	Inner Setting	Structural Characteristics—Physical Infrastructure	Risk of falling during corridor walking tests due to sudden door openings during the 6MWT	Warning signs during testing. Staff became used to exercise caution. Patients were guided to walk in the middle of the corridor. Over time, as the team became accustomed to the testing protocols and integrated themselves into routine processes, these cautionary measures were gradually phased out
December 2021	Inner Setting	Materials and Equipment	Concerns about risk of ankle injury	Stocked various ankle and knee orthoses and tape in a storage room. Acquired additional safety belts for HIT
December 2021	Individual Characteristics	Capability	Knowledge related to interpreting measurement results to support clinical decision-making	Laminated interpretation guides were placed at the test station. Results were discussed in team meetings to improve the clinicians’ understanding of patients’ progress and to encourage patients
December 2021	Individual Characteristics	Capability	Knowledge among physicians related to determining whether a high-intensity intervention is appropriate for a patient	Conducted meetings with PTs and medical doctors to clarify inclusion criteria and educate on how PTs monitor BP and intensity throughout treatment. Conferred with other hospitals and reviewed guidelines
Implementation of HIT 2022–2023				
Month/Year	CFIR Domain	CFIR Construct	Barrier Description	Implementation Strategy
2022–2023	Individual Characteristics	Capability	Lack of knowledge about how to deliver HIT to patients	Completing online courses on high-intensity gait training (HIT) to build fundamental knowledge
				Participating in workshops alongside more experienced PTs, facilitating practical skills
				Attending multi-site case meetings to share insights and align practices among different facilities
				Conducting weekly local meetings dedicated to case discussions, enabling continuous learning
				Utilizing video recordings of patient sessions to enhance case discussions and guide clinical decision-making
January 2022	Inner Setting	Materials and Equipment	Excessive time during treatment was used to locate braces	The storage room was moved close to the gym
January 2022	Individual Characteristics	Capability	Difficulty getting some patients in the zone because of orthopedic pain	Education and training on how to adapt HIT. Bodyweight support was used to help patients tolerate the training. Adjusted training speeds and varied tasks were used to maintain intensity
January 2022	Individual Characteristics	Capability	Difficulty sustaining intensity because of patients’ fatigue	Education and training on how to adapt HIT. Prioritized treatment with greater metabolic costs over shorter intervals
February 2022	Individual Characteristics	Capability	Lack of knowledge to provide HIT to students on the stroke team	Initially, students were assigned to other teams. After PTs gained HIT experience, they provided supervision for HIT sessions for some patients. Some students were introduced to a HIT education program depending on their interests and time
February 2022	Individual Characteristics	Capability	Remembering to document fidelity metrics daily	The documentation form and guide were discussed and co-developed to make documentation easier
April 2022	Individual Characteristics	Capability	Understanding how to challenge patients when they demonstrate symptoms such as dizziness, fatigue, nausea, and hypotension	Education and training on how to adapt HIT were provided. Treatment was modified to gradually increase a patient’s tolerance to HIT.
April 2022	Individual Characteristics	Capability	Understanding the clinical decision-making related to transitioning from HIT to usual care	Education and training on how to adapt HIT and clinical decision-making. A measurement interpretation guide during the evaluation aided in the interpretation of results. Findings were shared during team meetings to ensure a multidisciplinary approach. Updated guidelines for PTs included tailored treatments based on individual patient needs
April 2022	Inner Setting	Materials and Equipment	Limited availability of treadmill with harness system due to double booking	Coordination through shared timetables, rescheduling, or utilizing alternate equipment and times, such as early in the morning
April 2022	Individual Characteristics	Capability	Delayed medical clearance by physicians reduced the number of HIT sessions	Education was provided to appropriate patients for HIT. A process was also developed to determine appropriateness for HIT before initial consultations and discussions were held in team meetings, increasing the timeliness of HIT initiation
April 2022	Outer Setting	Local conditions	Length of stay	The length of stay was determined by the contract. An extension of the stay was considered when the patient continued to show improvements towards the end of the initial stay
August 2022	Inner Setting	Structural characteristics—work infrastructure	Time and priority	Team discussions resulted in consensus-based decisions. Treatment time for HIT was prioritized by the PT and team leader. Patients who benefited from HIT were distributed among different PTs. Patient goals were prioritized and determined the intervention focus
August 2022	Individual Characteristics	Capability	Technology competence. Efficiently monitoring HR, BP, and stepping metrics	PTs used paired HR monitors and smartphones. Internal education workshops improved competency in manual BP measurement. Guidelines for BP when undergoing HIT were locally adapted, and patient-specific restrictions were provided by the MDs when needed
August 2022	Inner Setting	Materials and Equipment	Arm bands for heart rate monitoring equipment (phones) were not fitting	New arm band holders were provided to each PT. The holders were placed on the forearm instead of the upper arm
August 2022	Individual Characteristics	Capability	Lack of knowledge related to the best clinical decision-making for HIT. The clinicians needed more practice with providing errors during treatment	PTs worked collaboratively during treatment, facilitating real-time decision-making discussions. The use of the HIT diagram showed the connection between skill acquisition and the need for assistance and error augmentation
September 2022	Innovation	Innovation Adaptability	Needed improved definitions for defining the target group and criteria for transitioning to usual care	Inclusion criteria were discussed with an MD or interdisciplinary team evaluating motivation, cognitive function, physical capabilities, as well as patient goals. The initial assessment was based on inclusion. Later evaluation was performed to reassess patients’ suitability for HIT
November 2022	Individual Characteristics	Capability	Clinicians felt they needed to improve decision-making related to maximizing variation while delivering HIT	PTs worked collaboratively during treatment, facilitating real-time decision-making discussions
January 2023	Individual Characteristics	Capability	Clinicians felt they needed to improve decision-making related to balancing HIT while addressing postural stability needs	Education was provided on the “sandwich-model”—alternating between high- and low-cost tasks to maintain intensity while addressing postural stability. Analyzing daily notes to assess patients’ responses to different tasks
March 2023	Individual Characteristics	Capability	Some patients with chronic stroke may have limited initial tolerance for HIT	Patients were gradually introduced to the intervention
March 2023	Individual Roles	Implementation Team Members	Hesitancy among MDs regarding BP guidelines for patients with hemorrhagic stroke	Local guidelines were adapted from general recommendations. Medical evaluations conducted by MDs to deter-mine patient eligibility for HIT were supplemented by direct feedback from physical therapists on patients’ blood pressure and heart rate responses during exercise. When necessary, initial moderate-intensity gait training was used as a starting point.
August 2023	Innovation	Innovation Complexity	Weekly reading and analyzing StepWatch	Reading and analyzing StepWatch data was occasionally conducted collaboratively to enhance proficiency among team members. Technical limitations often hindered the feasibility of performing detailed analyses during the patient’s stay. Education was provided on understanding the clinical value of maximizing daily stepping
August 2023	Individual Characteristics	Capability	Clinicians struggled with increasing the number of daily steps	Collaboration with nursing staff, especially for patients in need of assistance, was conducted. Education underscored the clinical value of increasing daily steps
September 2023	Individual Characteristics	Capability	Knowledge about monitoring HR in patients with atrial fibrillation	Education was provided about how to monitor patients with atrial fibrillation. The clinicians switched to chest monitors instead of arm monitors for HR monitoring, which improved accuracy
September 2023	Individual Characteristics	Capability	Hesitancy among PTs to use manual BP device	An automatic BP device was primarily used. Manual methods might provide more diagnostic value
September 2023	Individual Characteristics	Capability	Lack of knowledge and skills related to taking manual BP, resulting in increased time requirements for taking BP	Clinicians practiced manual BP procedures
Facilitators				
CFIR Domain	CFIR Construct	Description of Facilitator		
Implementation Process	Teaming	Participation in the implementation project was self-initiated by the team, as they expressed interest in joining voluntarily. It was not a top-down directive, but rather a self-driven decision to participate.		
Implementation Process	Teaming	Good collaboration between project participants, characterized by openness and mutual support.		
Individual Characteristics	Capability	Experienced physiotherapists, averaging >20 years of work experience.		
Individual Characteristics	Motivation	PTs demonstrated a strong motivation to follow evidence-based practices, driven by the goal to improve patient outcomes.		
Individual Roles	High-level leaders, Mid-level leaders	The team leader and department leader, both relatively new in their roles, were eager to engage in new projects and academic topics.		
Individual Roles	Implementation Leads	A dedicated project leader (team leader) was used with a strong focus on system, structure, and organization.		
Inner Setting	Information Technology Infrastructure	An existing system to manage and analyze data collection effectively.		
Inner Setting	Learning-Centeredness	Leadership actively supported participation in broader professional networks, with a desire to be actively involved in professional development and improve the quality of the existing rehabilitation program.		
Inner Setting	Learning-Centeredness	Leadership actively supported their participation in the project through meetings, workshops, and training, maintaining close clinical and practical involvement to understand the challenges that management could assist in resolving.		
Inner Setting	Learning-Centeredness	A genuine desire among the team members to improve and develop professionally.		
Inner Setting	Mission Alignment	A reputation for high-quality care is a priority for the leaders.		
Inner Setting	Relational Connections	Direct access to organizational decision-makers who provide support when needed or requested (financial requests—equipment, time, and required re-organization for the therapists to have time to work with the patients).		
Inner Setting	Relative Priority	With relatively few parallel projects running within the organization, it was possible to prioritize and allocate time for the project.		
Inner Setting	Structural Characteristics—Work Infrastructure	There was stability among the staff and a low turnover		
Innovation	Innovation Design	A clearly defined program with established fidelity metrics and outcome measurements.		
Outer Setting	Financing	Some funding was provided through Fysiofondet.		
Outer Setting	Local Attitudes	Leadership belief that providing high-quality care is an investment that results in increased patient referrals.		
Outer Setting	Partnerships and Connections	Clinical staff were involved in broader professional networks, with a desire for professional development and to contribute to improving the quality of rehabilitation services.		
Outer Setting	Partnerships and Connections	The Institute for Knowledge Translation and Regional Competence Service for Rehabilitation (RKR) provided valuable guidance and free access to resources.		
Outer Setting	Policies and Laws	The potential to elevate the quality of care was viewed as a critical factor in securing a renewed healthcare service contract.		

**Table 4:** Characteristics of the clinicians (n = 3).

Sex Male: n = 1 Female: n = 2
Age >40: n = 3
Years of practice >15: n = 3
Percentage of time on the stroke team 40%: n = 1 60%: n = 1 100%: n = 1
Number of patients with stroke seen daily 1–2: n = 1 3–4: n = 1 5–6: n = 1
Highest degree Bachelor’s = 3

**Table 5:** Coverage for HIT.

Coverage for HIT	2022	2023	2024	Total
Individuals admitted with stroke	126	128	17	271
Exclusion reasons				
Referred for other goals	37	34	8	79
Short stay	1	3	0	4
Admitted for constraint-induced movement therapy program	8	2	2	12
Medically unstable	2	5	0	7
Did not consent	1	1	0	2
Total excluded	49	45	10	104
Patients who may benefit from HIT	77	83	7	167
HIT was offered	39	46	2	87
Enrolled and completed FIRST project	30	30	1	61
Enrolled and dropped out	5	7	0	12
Previously enrolled in FIRST (data not collected at Skogli)	4	9	1	14
% coverage	51%	55%	29%	52%

## Data Availability

The original contributions presented in this study are included in the article/Supplementary Material. Further inquiries can be directed to the corresponding authors.

## Supplementary Materials

The following supporting information can be downloaded at: https://www.mdpi.com/article/10.3390/jcm14155409/s1, Table S1: Patient demographics and baseline measures; Table S2. (a) Fidelity Metrics in Patients with Subacute Stroke; (b) Fidelity Metrics in Patients with Chronic Stroke.
