# Supplementary material for: Implementing High-Intensity Gait Training in Stroke Rehabilitation: A Real-World Pragmatic Approach
Source: J Clin Med. 2025 Jul 31;14(15):5409. doi: 10.3390/jcm14155409 (PMC12347572; doi:10.3390/jcm14155409)
Supplement: Supplementary file 1 [file jcm-14-05409-s001.zip › jcm-3697701-supplementary.pdf]

Implementing High-Intensity Gait Training in Stroke Rehabilitation: A Real-World Pragmatic Approach

Jennifer L. Moore [1,2,*], Pia Krøll [3], Håvard Hansen Berg [3], Merethe B. Sinnes [3], Roger Arntsen [3], Chris E. Henderson [2,4], T. George Hornby [4], Stein Arne Rimehaug [1], Ingvild Lilleheie [1,5] and Anders Orpana [3]

Table S1.

Patient demographics and baseline measures							
**Subacute Stroke**							
	Age (years)	Time Post Stroke (days)	Length of Stay (days)	ADM SSV (m/s)	ADM FV (m/s)	6MWT ADM (m/s)	FAC ADM
Pre-Implementation (n=19)	71 (63-78)	40 (33-143)	28 (28-35)	.62 (.28)	0.92 (.38)	181 (94)	3 (2-4)
Implementation (n=8)	73 (67-74)	40 (12-99)	24 (21-28)	0.74 (.24)	.99 (.41)	225 (119)	4 (3-5)
Competency (n=13)	72 (66-80)	56 (33-67)	28 (28-35)	0.64 (.34)	1.04 (.48)	247 (121)	5 (3-5)
p-Value	0.76	0.81	0.08	0.65	0.61	0.27	0.07
**Chronic Stroke**							
	Age (years)	Time Post Stroke (days)	Length of Stay (days)	ADM SSV (m)	ADM FV (m)	6MWT ADM (m)	FAC ADM
Pre-Implementation (n=19)	71 (65-76)	454 (279-1623)	21 (21-24)	0.65 (.34)	0.89 (.43)	181 (103)	4 (4-5)
Implementation (n=22)	65.5 (54-74)	1084 (414-1634)	21 (21-28)	0.55 (.29)	0.90 (.36)	214 (113)	4 (4-5)
Competency (n=18)	67 (58-77)	1016 (636-2606)	21 (21-25)	0.72 (.36)	0.92 (.49)	235 (124)	4 (4-5)
p-Value	0.31	0.31	0.59	0.51	0.95	0.43	0.48

Table S2a. Fidelity Metrics in Patients with Subacute Stroke							
	Days of Stepping Data	Average Daily Stepping	Steps per Session	Min Stepping per session	Average Stepping Rate	Steps for 60 min sessions	Minutes Stepping for 60 min sessions
Pre-Implementation (phase 1, n=19)	13 (4)	3909 (2458)	748 (435-1044)	19 (6)	35 (9)	929 (565-1334)	23 (8)
Implementation (phase 2, n=8)	11 (4)	5542 (3353)	1853 (774-2195)	31 (11)	49 (17)	2312 (933-2821)	37 (12)
Competency (phase 3, n=13)	14 (3)	5033.5 (3038)	2306 (1492-2608)	37 (8)	54 (12)	2305 (1525-2784)	38 (8)
ANCOVA p-Value	0.09	0.41	<0.001	<0.001	<0.001	<0.001	0.023
Post-hoc p-Value	NA	NA	Phase 1-3: <0.001<br>Phase 1-2: 0.072<br>Phase 2-3: 0.600	Phase 1-3: <0.001<br>Phase 1-2: 0.003<br>Phase 2-3: 0.180	Phase 1-3: <0.001<br>Phase 1-2: 0.025<br>Phase 2-3: 0.628	Phase 1-3: 0.001<br>Phase 1-2: 0.035<br>Phase 2-3: 1.000	Phase 1-3: 0.031<br>Phase 1-2: 0.120<br>Phase 2-3: 0.958

	Days of HR data	Session Duration (Min)	HR Max	HR Avg	Time in HR Zone absolute	% session in the target HR Zone	RPE Max	Time in RPE Zone absolute	% session in the target RPE Zone
Pre-Implementation (phase 1, n=19)	13 (4)	40 (6)	67% (8%)	54% (7%)	1 (0-3)	1% (0-6%)	14 (13-14)	2 (0-7)	5% (1-15%)
Implementation (phase 2, n=8)	11 (4)	41 (5)	77% (12%)	65% (11%)	18 (3-28)	40% (6-56%)	16 (14-18)	22 (5-26)	48% (12-63%)
Competency (phase 3, n=13)	14 (3)	44 (4)	77% (9%)	64% (8%)	20 (4-26)	45% (10-54%)	17 (16-17)	21 (6-31)	34% (28-51%)

p-Value	0.091	0.238	0.005	0.003	0.001	0.002	<0.001	<0.001	<0.001
post-hoc p-value	NA	Phase 1-3: <0.001<br>Phase 1-2: 0.003<br>Phase 2-3: <0.001	Phase 1-3: 0.010<br>Phase 1-2: 0.029<br>Phase 2-3: 1.000	Phase 1-3: 0.012<br>Phase 1-2: 0.012<br>Phase 2-3: 0.906	Phase 1-3: 0.001<br>Phase 1-2: 0.005<br>Phase 2-3: 0.973	Phase 1-3: 0.003<br>Phase 1-2: 0.013<br>Phase 2-3: 1.000	Phase 1-3: 0.000<br>Phase 1-2: 0.017<br>Phase 2-3: 0.206	Phase 1-3: <0.001<br>Phase 1-2: 0.003<br>Phase 2-3: 1.000	Phase 1-3: <0.001<br>Phase 1-2: 0.001<br>Phase 2-3: 1.000

Table S2b. Fidelity Metrics in Patients with Chronic Stroke

	Number of Days Included	Average Daily Stepping	Steps per Session	Min Stepping per session	Average Stepping Rate	Steps for 60 min sessions	Minutes Stepping for 60 min sessions
Pre-Implementation (n=19)	9 (8-10)	3678 (2502)	584 (280 - 809)	16 (12-19)	32 (11)	749 (418)	19 (7)
Implementation (n=20)	10 (9-13)	5191 (3226)	1803 (1130-2155)	33 (26-37)	48 (12)	1913 (695)	37 (7)
Competency (n=18)	10 (10-11)	5758 (2562)	2001 (1467-2552)	38 (29-43)	52 (15)	2192 (947)	39 (9)
p-value	0.946	0.07	<0.001	<0.001	<0.001	<0.001	<0.001
post-hoc p-value	NA	NA	Phase 1-3: <0.001<br>Phase 1-2: <0.001<br>Phase 2-3: 0.140	Phase 1-3: <0.001<br>Phase 1-2: <0.001<br>Phase 2-3: 0.073	Phase 1-3: <0.001<br>Phase 1-2: 0.006<br>Phase 2-3: 0.592	Phase 1-3: <0.001<br>Phase 1-2: <0.001<br>Phase 2-3: 0.450	Phase 1-3: <0.001<br>Phase 1-2: <0.001<br>Phase 2-3: 0.770

	Days of HR data	Session Duratio n (Min)	HR Max	HR Avg	Time in HR Zone absolute	% session in the target HR Zone	RPE Max	Time in RPE Zone absolute	% session in the target RPE Zone
Pre-Implementation (n=19)	9 (1)	45 (45-47)	71% (8%)	58% (54-64%)	2 (1-6)	5% (1-13%)	3 (1-7)	3 (1-7)	6% (2%-16%)
Implementation (n=20)	11 (3)	53 (49-56)	80% (5%)	65% (62-69%)	17 (11-26)	31% (20-53%)	16 (15-17)	19 (16-25)	37% (28-47%)
Competency (n=18)	11 (2)	56 (54-59)	80% (8%)	64% (58-71%)	13 (8-31)	21% (15-55%)	16 (15-17)	20 (16-27)	36% (29-50%)
p-value	0.006	<0.001	<0.001	0.007	<0.001	<0.001	<0.001	<0.001	<0.001
post-hoc p-value	Phase 1-3: 0.050<br><br>Phase 1-2: 0.006<br><br>Phase 2-3: 0.770	Phase 1-3: <0.001<br><br>Phase 1-2: <0.001<br><br>Phase 2-3: 0.003	Phase 1-3: 0.001<br><br>Phase 1-2: 0.001<br><br>Phase 2-3: 0.975	Phase 1-3: 0.014<br><br>Phase 1-2: 0.003<br><br>Phase 2-3: 1.000	Phase 1-3: <0.001<br><br>Phase 1-2: <0.001<br><br>Phase 2-3: 0.581	Phase 1-3: <0.001<br><br>Phase 1-2: <0.001<br><br>Phase 2-3: 0.396	Phase 1-3: <0.001<br><br>Phase 1-2: <0.001<br><br>Phase 2-3: 0.411	Phase 1-3: <0.001<br><br>Phase 1-2: <0.001<br><br>Phase 2-3: 0.411	Phase 1-3: <0.001<br><br>Phase 1-2: <0.001<br><br>Phase 2-3: 0.840
